# Autophagy and senescence facilitate the development of antiestrogen resistance in ER positive breast cancer

**DOI:** 10.3389/fendo.2024.1298423

**Published:** 2024-03-18

**Authors:** Michael K. McGrath, Ali Abolhassani, Luke Guy, Ahmed M. Elshazly, John T. Barrett, Nahid F. Mivechi, David A. Gewirtz, Patricia V. Schoenlein

**Affiliations:** ^1^ Georgia Cancer Center, Augusta University, Augusta, GA, United States; ^2^ Department of Cellular Biology & Anatomy, Medical College of Georgia at Augusta University, Augusta, GA, United States; ^3^ Department of Pharmacology & Toxicology, Virginia Commonwealth University, Richmond, VA, United States; ^4^ Massey Comprehensive Cancer Center, Virginia Commonwealth University, Richmond, VA, United States; ^5^ Department of Pharmacology and Toxicology, Faculty of Pharmacy, Kafrelsheikh University, Kafrelsheikh, Egypt; ^6^ Department of Radiation Oncology, Georgia Cancer Center, Medical College of Georgia at Augusta University, Augusta, GA, United States

**Keywords:** autophagy, senescence, endocrine resistance, breast cancer, SERMs, SERDs, aromatase inhibitors, CDK4/6 inhibitors

## Abstract

Estrogen receptor positive (ER^+^) breast cancer is the most common breast cancer diagnosed annually in the US with endocrine-based therapy as standard-of-care for this breast cancer subtype. Endocrine therapy includes treatment with antiestrogens, such as selective estrogen receptor modulators (SERMs), selective estrogen receptor downregulators (SERDs), and aromatase inhibitors (AIs). Despite the appreciable remission achievable with these treatments, a substantial cohort of women will experience primary tumor recurrence, subsequent metastasis, and eventual death due to their disease. In these cases, the breast cancer cells have become resistant to endocrine therapy, with endocrine resistance identified as the major obstacle to the medical oncologist and patient. To combat the development of endocrine resistance, the treatment options for ER^+^, HER2 negative breast cancer now include CDK4/6 inhibitors used as adjuvants to antiestrogen treatment. In addition to the dysregulated activity of CDK4/6, a plethora of genetic and biochemical mechanisms have been identified that contribute to endocrine resistance. These mechanisms, which have been identified by lab-based studies utilizing appropriate cell and animal models of breast cancer, and by clinical studies in which gene expression profiles identify candidate endocrine resistance genes, are the subject of this review. In addition, we will discuss molecular targeting strategies now utilized in conjunction with endocrine therapy to combat the development of resistance or target resistant breast cancer cells. Of approaches currently being explored to improve endocrine treatment efficacy and patient outcome, two adaptive cell survival mechanisms, autophagy, and “reversible” senescence, are considered molecular targets. Autophagy and/or senescence induction have been identified in response to most antiestrogen treatments currently being used for the treatment of ER^+^ breast cancer and are often induced in response to CDK4/6 inhibitors. Unfortunately, effective strategies to target these cell survival pathways have not yet been successfully developed. Thus, there is an urgent need for the continued interrogation of autophagy and “reversible” senescence in clinically relevant breast cancer models with the long-term goal of identifying new molecular targets for improved treatment of ER^+^ breast cancer.

## 1 Introduction

It is estimated that in 2023, in the United States over 275,000 cases of invasive breast cancer will be diagnosed in women and over 2,500 cases in men (1). Of these breast cancers, approximately 70% are diagnosed as hormone receptor positive (HR^+^) based on detectable expression levels of the estrogen receptor α (ERα) and/or progesterone receptor (PR) (2, 3). HR^+^ breast cancer cell growth and viability typically shows a dependence on ER function, which allows treatment options with targeted endocrine therapies, also referred to as hormone or antiestrogen therapy. There are two main subgroups of HR^+^ breast cancer, Luminal A and Luminal B (4). The Luminal B breast cancers typically express higher levels of the proliferation marker, Ki67 (5), show increased metastasis to lymph nodes, and have a worse prognosis and a greater chance for local recurrence and survival than patients with Luminal A subtype (6). HER2/Neu positive breast cancers comprise approximately 20% of breast cancer cases that present annually (7). HER2/Neu, the human epidermal growth factor-2 receptor, also referred to as Erb-b2, is gene amplified and overexpressed in this subset of cancers which are not typically sensitive to endocrine therapy (8, 9). Both small molecules and/or antibodies have been developed which target and inhibit HER2/neu function. Triple negative breast cancer (TNBC), also referred to as basal breast cancer, has the lowest occurrence rate (approximately 15-17%) of breast cancers, but is highly aggressive with metastases identified in nearly half of all patients (10). TNBCs do not express ERα or PR and do not have gene amplification of HER2/Neu. Further, TNBC are highly heterogeneous, with at least six described subgroups (11, 12). Because a molecular target has not been identified to allow targeted therapy, chemotherapy is the standard treatment option for TNBC.

Overall, on an annual basis, breast cancer is the second leading cause of cancer deaths in woman, with greater than 40,000 deaths annually (1). Most of these deaths occur in patients initially diagnosed with HR^+^ breast cancer who succumb to their disease after multiple iterations of endocrine therapies plus adjuvants such as CDK4/6 inhibitors or chemotherapy. In large part, the mortality of HR^+^ breast cancer is due to endocrine resistance. Overall, it is estimated that 30 to 40 percent of HR^+^ breast cancer patients will express endocrine resistance at some point during their treatment (13). In this chapter, we discuss the multiple mechanisms of hormone resistance, referred to as antiestrogen resistance throughout, with a focus on autophagy and senescence as survival modes that precede the development of genetic mutations or epigenetic changes in breast cancer cells required for escape from cell cycle arrest, estrogen dependency, and dissemination (metastasis). We further discuss the concept that acquired antiestrogen resistance would be highly reduced if the first line treatments for HR^+^ breast cancer included a sequential regimen during which effective autophagy inhibitors and/or senolytic agents that eliminate senescent cells were administered to patients after breast cancer cells entered these cell survival modes.

## 2 Antiestrogen therapies to target ER function

A diagnosis of HR^+^ breast cancer is based on detectable expression of the ERα in a minimum of 1% of the cells of the tumor biopsy (14). ERα expression is strongly associated with a dependence of breast cancer cells on estrogen for growth, although breast cancers with low ER expression (1-10% of total tumor cells) may grow independently of estrogen (14). PR expression is also analyzed and typically is detected in ER^+^ breast cancers. The *PgR* gene encodes PRA and PRB, two progesterone receptor isoforms, and *PgR* transcription is regulated by estrogen-bound ERα (15). However, PR expression in the absence of ERα expression can occur and is seen in a small subset of HR^+^ breast cancers that are typically more aggressive than luminal A or B breast cancer and typically not sensitive to antiestrogen treatment (16). Whether targeting PR in these breast cancers would provide an effective treatment has not been determined. Notably, PR antagonists are available (17), and a recent clinical trial (MIPRA; NCT02651844) provided evidence that the progesterone antagonist, mifepristone (RU486), may provide benefits in ER^+^ breast cancer expressing a high PRA/PRB isoform ratio; an approximately 50% decrease in Ki67 staining was observed in all surgical specimens from patients treated with mifepristone compared with baseline (P = 0.0003) (18). The authors further proposed that “the combined effects of mifepristone and estrogen receptor modulators warrant clinical evaluation to improve endocrine treatment responsiveness in these patients.” This recent report supports earlier studies (19–21), including pre-clinical studies from our laboratory (22, 23), that proposed the combined use of an antiestrogen and the antiprogestin, mifepristone, to more effectively kill breast cancer cells and circumvent the development of antiestrogen resistance in ER^+^ breast cancer. Further, a study by the Shapiro laboratory showed that progestin stimulates the proliferation of breast cancer cells harboring mutant ERα that reduced sensitivity to antiestrogen treatments (24), providing further support to an antiprogestin treatment regimen. Estrogen receptor β, a second estrogen receptor whose function in breast cancer is not well understood (25), is not routinely analyzed by pathologists.

### 2.1 SERDs, SERMs, and AIs

In the clinical setting, antiestrogens used to target the ERα include selective estrogen receptor modulators (SERMs), and selective estrogen receptor downregulators (SERDs) (3). SERMs and SERDs bind to ERα and block the binding of estrogen to this receptor. The estrogen bound ERα enters the nucleus and functions as a transcription factor binding to estrogen response elements (EREs) in the promoter regions of more than two-hundred genes, including the promoter of the *PgR*. Binding to EREs drives increased transcription of genes required for cell cycle progression in normal cells involved in female reproduction (26), as well as in breast cancer cells (27). ERα-mediated transcription and the role of accessory co-activators and co-repressors in normal and breast cancer cells has been reviewed recently (28). The binding of SERMs and SERDs inhibits estrogen mediated ERα function/signaling in breast cancer cells (29). In addition, the binding of a SERD promotes ERα degradation in the proteasome.

Although tamoxifen has been the most frequently utilized antiestrogen for decades, tamoxifen also acts as an agonist in some tissues such as the endometrium and can cause endometrial pathologies, including cancer in a small cohort of patients (30). Thus, fulvestrant and other SERDs may be favored in the clinical setting and are commonly used to treat the pre-menopausal breast cancer patient (29). For post-menopausal women, aromatase inhibitors (AIs) are becoming the preferred standard-of-care. These inhibitors block the production of estrogen via the aromatase enzyme. Aromatase converts androstenedione, testosterone, and 16α-hydroxysterone into estrone, estradiol, and estriol, respectively (31). Currently, three third-generation AIs are regularly administered as primary therapies for HR^+^ breast cancer, namely two nonsteroidal derivatives, anastrozole (Ana) and letrozole (Let), and one steroidal derivative, exemestane (Exe) (32). AIs target the human aromatase enzyme, a member of the cytochrome P450 family, encoded by the *CYP19A1* gene on chromosome 15 (33),. Aromatase expression is present in organs, such as endometrium, bone, brain, and adipose tissue. In addition, breast cancer cells themselves can express aromatase (34) or affect cells in the microenvironment such as adipocytes to upregulate aromatase expression (35).

### 2.2 Sequential antiestrogen therapy in the management of ER+ HER2 negative breast cancer

The use of antiestrogens is a highly effective treatment for ER^+^ breast cancers (36). However, approximately 30% of breast cancers show intrinsic (primary resistance) or acquire resistance (secondary resistance) to antiestrogen treatments. Intrinsic resistance is recognized early in treatment as the breast cancer cells ability to continue to proliferate during therapy. Acquired resistance occurs during treatment, after an initial period of breast cancer sensitivity to the antiestrogen treatment. In either case, resistance to endocrine therapy is a major clinical challenge and understanding the molecular mechanisms of intrinsic and acquired resistance are required to improve treatment outcomes.

**Figure 1:**
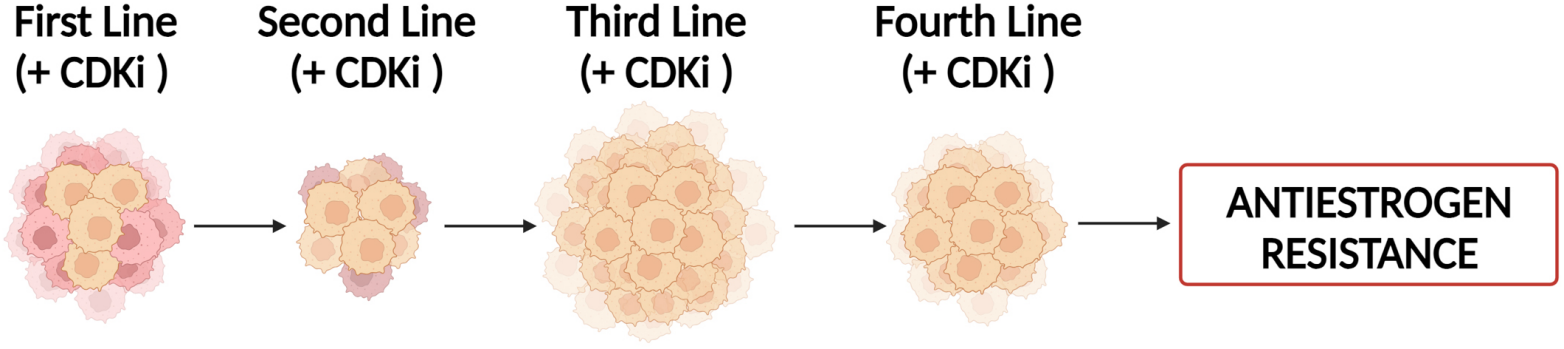
Sequential antiestrogen treatment (AE) in the management of breast cancer, with a CDKi, used as an adjuvant to delay antiestrogen resistance. As a first line therapy, ER^+^ breast cancer cells treated with either a SERM, SERD, or AI, develop antiestrogen resistance. The antiestrogen-resistant, ER^+^ breast cancer cells are then treated with a different SERM or SERD. This sequential treatment with different antiestrogens delivered as 3^rd^ and 4^th^ line therapies delays relapse, particularly if a CDKi is utilized with the antiestrogen. Antiestrogen resistance potentially results from a pre-existing population of resistant breast cancer cells in the primary heterogeneous tumor cell population (depicted in yellow) or a subpopulation of breast cancer cells that acquire resistance via genetic or epigenetic events.

A common approach to treating antiestrogen resistant breast cancers is to change the antiestrogen being used for treatment. This approach is often effective because resistance to one antiestrogen does not necessarily impart cross-resistance (resistance to other antiestrogens). For example, breast cancer cells resistant to aromatase inhibitors can be successfully treated with fulvestrant (37). This sequential approach of switching antiestrogens to combat resistance (schematically depicted in **Figure 1**) is successful in the management of breast cancer, but often falls short in curing the disease, and relapse is typically inevitable.

The recognition that breast cancer eradication will require an understanding of the endocrine resistance mechanisms has led to innumerable lab-based studies (38, 39) to establish and analyze antiestrogen and aromatase inhibitor resistant breast cancer models. These studies have resulted in the identification of a plethora of resistance mechanisms as well as multiple molecular targets (discussed in Section 7), with the targeting of CDK4/6 (discussed below, Section 3) being one of the most successful improvements in the treatment of ER^+^, HER2/NEU negative breast cancer (40–42). This combined therapy approach significantly delays relapse; however, resistance can occur even when antiestrogens are combined with a CDK4/6 inhibitor (CDKi) (43–45)

## 3 CDKi as an adjuvant to antiestrogen therapy

Basic research identifying the role of the cyclin D-CDK4/6-Rb axis in G1 to S cell cycle transit, followed by translational and clinical studies in which this axis was linked to breast cancer progression and antiestrogen resistance (46, 47), culminated in the development of CDKi’s currently used in combination with endocrine treatments for ER^+^ HER2 negative breast cancer (48). CDK4/6 small molecule inhibitors currently used as an adjuvant to antiestrogen treatment include palbociclib, ribociclib, and abemaciclib. These molecules selectively target CDK4/6 and activate the retinoblastoma protein (Rb), a tumor suppressor that binds E2F transcription factors and halts the cell cycle in the G1 phase. The efficacy of these inhibitors has been demonstrated in multiple solid tumors, including breast cancer (40). In the initial Phase II (PALOMA-1) and III (PALOMA-2-and -3) trials, palbociclib in combination with fulvestrant or letrozole demonstrated superior efficacy for advanced ER^+^ breast cancer compared to either antiestrogen used as a single agent treatment, with a doubling of progression-free survival (41, 42); increased median progression-free survival (PFS) of up to 27.6 months for letrozole + palbociclib compared to 14.5 months for letrozole as a single agent. An improved response has also been demonstrated when combining aromatase inhibition with abemaciclib as well as ribociclib (49). Unfortunately, either intrinsic or acquired resistance occurred in approximately 15-20% of ER^+^ HER2 negative breast cancer patients (50), with adverse effects in some patients, such as neutropenia in response to ribociclib and palbociclib (51). Currently, there are a minimum of eleven active clinical trials in the United States further evaluating CDKi’s combined with endocrine therapy for ER^+^ HER2 negative breast cancer (**Table 1**).

**Table 1:** List of active clinical trials of CDK inhibitor in the U.S.

NCT (Study Name)	Intervention	Phase	Primary Endpoint
NCT03285412: CDK 4/​6i, Ribociclib, With Adjuvant Endocrine Therapy for ER-positive Breast Cancer (LEADER)	Ribo + ET	II	Evaluate Ribo efficacy in patients with MRD based on ctDNA.
NCT01709370: Letrozole and CDKi Inhibitor for ER Positive, HER2 Negative Breast Cancer in Postmenopausal Women	Pal + Let, Ribo + Let, Abe + Let	II	A head-to-head comparison of the three CDK4/6i immune modulation.
NCT05766410: A Randomized Study Comparing the Immune Modulation Effect of Ribociclib, Palbociclib, and Abemaciclib in ER+/​HER2- EBC (ORACLE-RIPA)	Pal + G1T48 (SERD) vs. G1T48 alone	I	Investigate the clinical benefit of G1T48 ± Pal.
NCT02917005: A Phase II Study of Ovarian Function Suppression and Exemestane with or without Palbociclib in Premenopausal Women with ER Positive/HER-2 Negative Metastatic Breast Cancer	Pal + Exe + OFS (Arm 1) or Exe +OFS (Arm 2)	II	Evaluate effects of Pal + Exe + ovarian function suppression (OFS) versus OFS plus Exe.
NCT05293964: A Multicenter, Open-label, Phase I Clinical Study to Evaluate the Safety, Pharmacokinetics, and Antitumor Efficacy of SIM0270 Alone or in Combination in Subjects With ER-positive, HER-2 Negative Locally Advanced or Metastatic Breast Cancer	SIM0270 (SERD)+ Pal or everolimus (mTOR inhibitor)	Ia + Ib	Ia- Evaluate the safety and efficacy of SIM0270. Ib- Evaluate dose expansion, escalation, safety, and efficacy of SIM0270 single agent or in combination with Pal ± everolimus.
NCT02297438: A Study of Palbociclib (PD-0332991) + Letrozole VS. Placebo+ Letrozole For 1st Line Treatment of Asian Postmenopausal Women With ER+/​HER2- Advanced Breast Cancer (PALOMA-4)	Pal + Let vs Let + Placebo	III	Compare the clinical benefit of Let + Pal versus Let + placebo
NCT05262400: A Study to Learn About the Study Medicine (Called PF-07220060 in Combination with PF-07104091) in Participants with Breast Cancer and Solid Tumors	PF-07220060 + PF-07104091 +/- Ful or Let	I + II	Evaluate safety and efficacy of the CDKi study medicines PF-07220060 and PF-07104091.
NCT01723774: PD 0332991 and Anastrozole for Stage 2 or 3 Estrogen Receptor Positive and HER2 Negative Breast Cancer	Pal (PD0332991), Ana, Goserelin	II	To investigate the utility of Pal + Ana to treat early-stage ER+/HER2- breast cancer
NCT05305924: Fulvestrant+Abemaciclib With or Without Run-In of Fulvestrant in ER-Positive, Her2-Negative Metastatic Breast Cancer	Ful + Abe with vs without run-in Ful	II	Comparing time to treatment failure for Ful plus Abe with or without 1-month run in of Ful.
NCT04964934: Phase III Study to Assess AZD9833+ CDK4/6i in HR+/HER2-MBC With Detectable ESR1m Before Progression (**SERENA-6**)	AZD9833, AZD9833 placebo, Ana, Ana placebo, Let, Let placebo, Pal, Abe, LHRH agonist, Ribo	III	Comparing AZD9833 plus Pal, Abe, or Ribo compared to Ana/Let plus Pal, Abe, or Ribo to see if this new drug works better than AIs with CDKi.
NCT03054363: Tucatinib, Palbociclib and Letrozole in Metastatic Hormone Receptor Positive and HER2-positiveBreast Cancer	Tucatinib + Pal + Let	I and II	Measuring the safety and tolerability of Tucatinib in combination with Pal and Let.

NCT, ClinicalTrials.gov identifier; AI, aromatase inhibitor; CDKi, CDK 4/6 inhibitors; SERD, selective receptor degrader; Pal, palbociclib; Ribo, ribociclib; Abe, abemaciclib; Let, letrozole; Ful, fulvestrant; Exe, exemestane; OFS, ovarian function suppression, leuprolide; LHRH, luteinizing hormone-releasing hormone; ET, endocrine therapy; MRD, minimal residual disease; Ana, anastrozole, ctDNA, circulating tumor DNA; mTOR, mammalian target of rapamycin.

### 3.1 CDKi resistance and autophagy

Resistance to a CDKi such as palbociclib does not necessarily predict resistance to ribociclib or abemaciclib, allowing a different CDKi to be incorporated in the treatment regimen. To date there are neither biomarkers to identify which patients will derive optimal benefit from a particular CDKi, nor expression profiles to identify breast cancer cells that express intrinsic resistance or are more likely to acquire CDKi resistance. However, studies by Vijayaraghavan et al. identified induction of autophagy and senescence by the CDKi palbociclib (52). Autophagy is a highly conserved catabolic pathway in normal and cancer cells that can be induced above basal levels in response to a multitude of stresses including nutrient deprivation during which macromolecules (substrates) are recycled to sustain cellular homeostasis (53, 54). Of particular interest, the combined treatment of palbociclib + autophagy inhibitors (small molecules and genetic approaches) synergistically induced senescence in Rb positive, cytoplasmic cyclin E negative breast cancers (52). Thus, in this experimental system, autophagy appeared to protect cells from senescence, although this is not uniformly the case for these responses (55–57). The senescence induced by the autophagy inhibitors appeared to be an irreversible state of growth arrest, at least for the duration of the study. However, it was not determined whether the senescent cells expressed the senescence associated secretory phenotype (SASP) (58), that has been associated with tumor relapse and tumor progression (metastasis) in a mouse model of breast cancer (59).

## 4 Autophagy: a potential molecular target for ER+ breast cancer

### 4.1 Types of autophagy

Autophagy is an intracellular pathway for the catabolic degradation of cytoplasmic (damaged and/or misfolded) proteins and organelles during which the resulting macromolecules are recycled to provide energy and support essential cellular functions (60, 61). As a routine housekeeping role, autophagy removes aggregated proteins, damaged organelles, and pathogens from cells (62). There are three commonly accepted forms of autophagy: macroautophagy, microautophagy, and chaperone-mediated autophagy (CMA) (62). Each mode of autophagy shares the ultimate goal of catabolizing cellular contents (e.g., proteins and organelles that have become damaged or aged), which in turn delivers requisite macromolecules for cellular function (63). These autophagy processes are induced by various stressors (e.g., hypoxia, starvation, or infection) (63). Microautophagy is characterized by lysosomal engulfment of intracellular substrates in small quantities via membranous invagination (64). CMA relies on heat shock proteins (namely HSC70 or HSPA8) and co-chaperones to target substrates containing a recognizable KFERQ motif for translocation into the lysosome through lysosomal-associated membrane protein 2A (LAMP-2A) (65). Macroautophagy non-selectively catabolizes bulk cytoplasmic contents via membrane engulfment or is selective in its catabolism depending on the recruitment by a receptor protein such as p62 (also known as SQSTM1), NBR1, optineurin (OPTN), NDP52 which simultaneously bind cargo and ubiquitin and contribute to autophagy initiation and membrane recruitment. There are a plethora of selective autophagy pathways and autophagy receptors detailed in a recent review (66).

**Figure 2:**
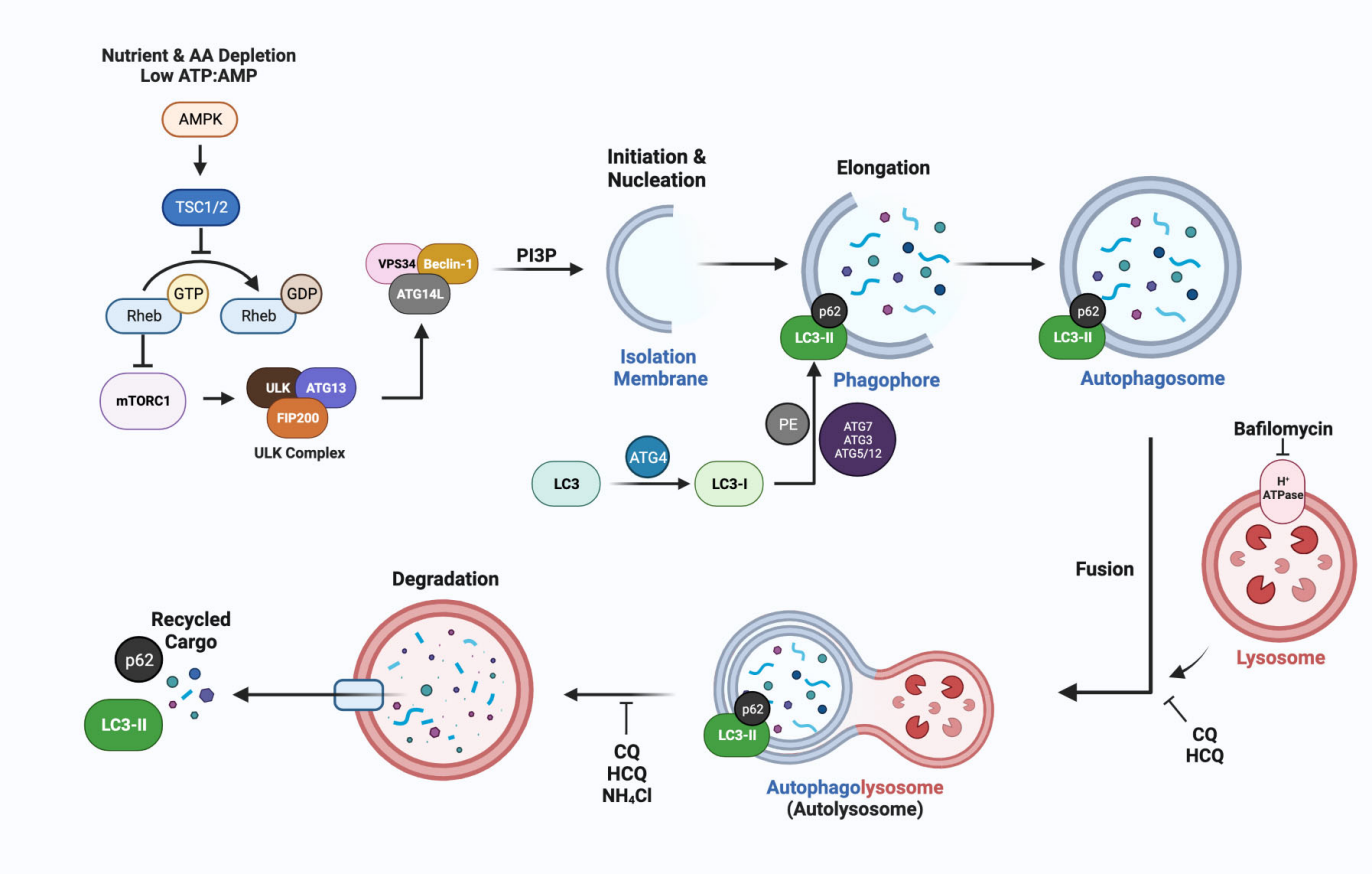
A simplified schematic of the molecular mechanisms of autophagy, emphasizing steps in the process that can be inhibited by lysomotrophic agents such as chloroquine (CQ), hydroxychloroquine (HCQ), the ATPase inhibitor bafilomycin, and NH_4_Cl. The most widely used markers for autophagy are LC3-II generation and p62/SQSTM1 degradation which can be measured using immunoblotting or immunofluorescence approaches to quantify autophagic flux (68). ATG-encoded proteins provide additional targets for inhibition at nearly every step of autophagosome formation and maturation. The lysomotropic agents hydroxychloroquine (HCQ) or chloroquine (CQ), that disrupt the acidic pH required for autolysosomal turnover and/or directly block fusion between the lysosome and autophagosome are FDA approved and are being used in breast cancer clinical trials (Section 5, **Table 2**).

The non-selective pathway of macroautophagy, designated autophagy throughout this review, has been implicated in the development of endocrine resistance and its regulation and function is dependent on autophagy related genes, designated *ATG.* A recent review provides an in-depth discussion of *ATG* genes and their respective functions in autophagosome formation and function (67). **Figure 2** is a brief synopsis detailing the basic core machinery of the autophagy pathway, which is similarly utilized for selective and non-selective autophagy, is essential in regulating cellular energy homeostasis, and can be induced above basal physiological levels to combat stresses such as nutrient deprivation, hypoxia, radiation, chemotherapy, and endocrine therapies.

### 4.2 Phagophore initiation, elongation, and closure

Mammalian autophagy begins with the formation of a phagophore (**Figure 2**); however, the origins and corresponding contexts from which the membranous contents are derived remain contested. Current studies illustrate reproducible contributions from the endoplasmic reticulum (ER), trans-Golgi network (TGN), late endosomes, and the plasma membrane (PM) (69). The relatively low transmembrane protein character within these structures implies a possibility for *de novo* synthesis using cytosolic lipids (70). The initiation step is regulated by the mammalian target of rapamycin complex (mTORC1), which is sensitive to rapamycin inhibition, and senses both nutrient deprivation and amino acid scarcity (71). In response to these cellular stresses, unc-51-like autophagy activating kinase (ULK1/2) dissociates from mTORC1 after a dephosphorylation event. Subsequently, ULK1/2 undergoes autophosphorylation, and phosphorylates ATG13 and FIP200 (which are recruited to the ULK1/2 complex). This phosphorylation cascade continues when the ULK1/2 complex interacts with the class III PI3K complex members (AMBRA1, BECN1, VPS15/34, UVRAG, ATG14, and NRBF2), which are primarily responsible for phagophore nucleation during which the complex binds to membrane, and generates PI (1)P in a VPS34-dependent manner (72).

Elongation of the phagophore is conducted via sequential steps mediated by ATG proteins. For instance, the ATG5/12 complex (conjugated by ATG16L) plays a critical role in elongation, as do MAP1LC3B, the microtubule-associated proteins 1A/1B light chain 3B (herein denoted as LC3) which is encoded by *ATG8* (73). In an initial step, cytosolic LC3-I is conjugated with phosphatidylethanolamine (PE) in a process dependent on ATG4, ATG7, ATG3, and the ATG5/12 complex. During this process, LC3-I is cleaved by ATG4(B) to expose a C-terminal glycine residue, required for the PE conjugation. The lipidated form, LC3-II can directly bind p62/SQSTM1 and other receptor proteins, via their LC3- interacting region (LIR) to bring “select” cargo such as ubiquitinated proteins to the autophagosome.

### 4.3 Autophagolysosome formation and flux

Once the phagophore has been expanded and processed into the autophagosome structure, its hydrolytic activity is dependent upon its fusion with a lysosomal body; the fusion generates the autophagolysosome (more simply, the autolysosome). This fusion is mediated by SNARE proteins and features syntaxin 17 (STX17), synaptosomal-associated protein 29 (SNAP29) and vesicle-associated membrane protein 8 (VAMP8) (74). Coinciding with the fusion of the autolysosome is its acidification that activates a multitude of hydrolytic enzymes, including cathepsin L and B that are involved in the degradation of all engulfed cargo and the autolysosome itself, with turnover of LC3-II and associated receptor proteins such as p62.

### 4.4 Stimulus-induced autophagy: mTORC1 and mTORC2

**Figure 3:**
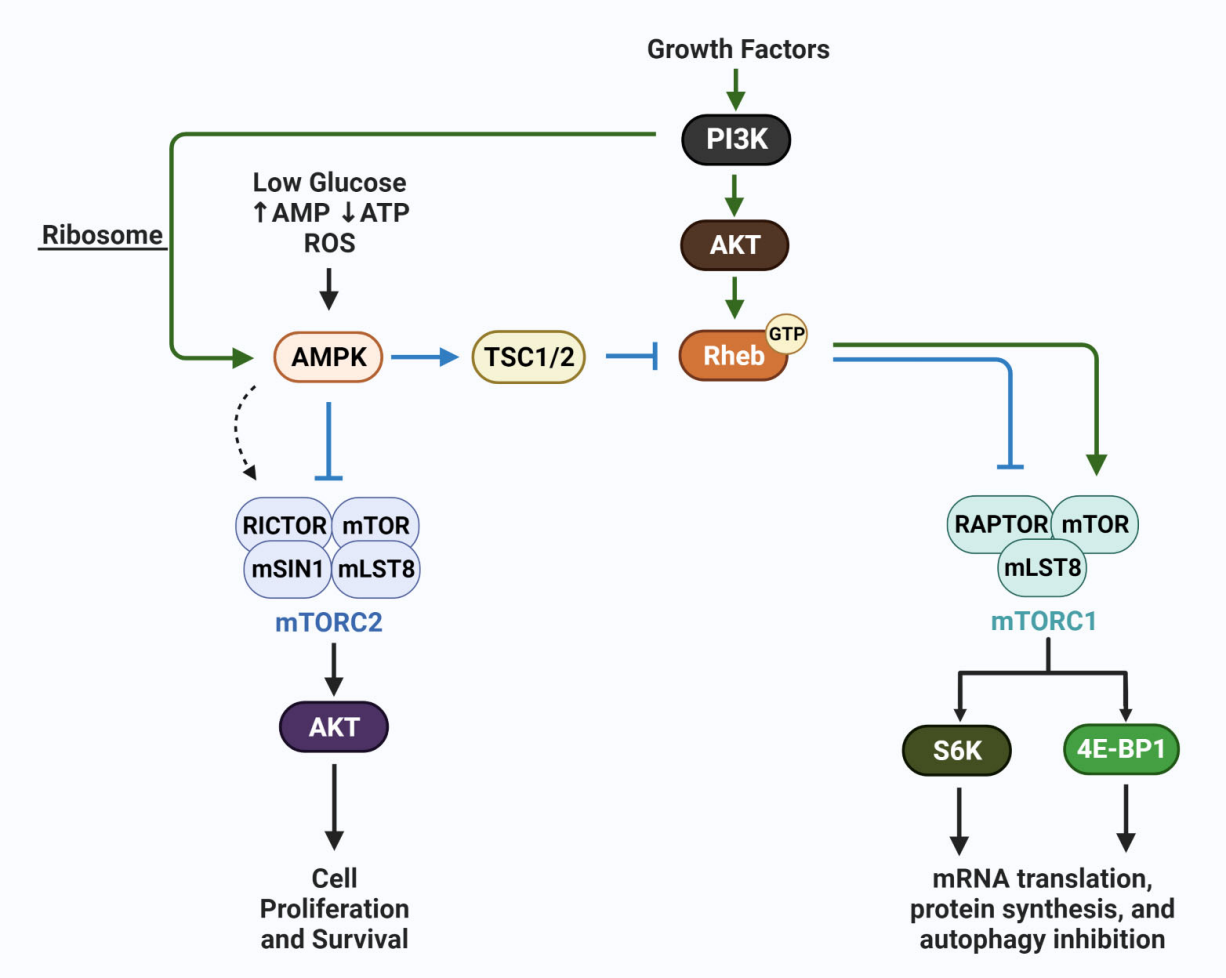
AMPK regulation of mTORC1 and mTORC2. The schematic shows growth factor-mediated stimulation of mTORC1 (via Rheb) and mTORC2 (via PI3K/ribosome interaction) (green arrows), and the canonical action of AMPK which is to inhibit mTORC1 and mTORC2. In contrast, under conditions of energetic stress, AMPK has been reported to selectively activate mTORC2 (dotted arrow); mTORC2 subsequently phosphorylates AKT and promotes cell survival. These actions of AMPK, members of the mTOR complexes, including mLST8, and mSIN1, upstream activators i.e. GTP:RHEB, and the downstream effectors S6K and 4E-BP1 are discussed in detail in recent reviews (76, 78).

In addition to mTORC1, a second complex mTORC2, which is not sensitive to rapamycin inhibition, is involved in the regulation of autophagy (75). These complexes are key conduits that regulate extracellular growth signals with intracellular metabolic processes, with overlap in function between mTORC1 and mTORC2. These two complexes are key regulators of autophagy levels in cells, with autophagic recycling of macromolecules typically inhibited when mTORC1 and mTORC2 are active. In response to nutrient availability and energy levels, mTORC2 along with mTORC1, activate multiple substrates that regulate cellular mass via protein, nucleic acid and lipid production, glucose metabolism, and mitochondrial metabolism (76). The mTORC1 complex typically responds to nutrient availability by regulating protein synthesis via 4E-BP1 and S6K, while mTORC2 is sensitive to growth factor availability and PI3K. The mTORC1 and mTORC2 complexes have overlapping functions and regulate each other to amplify responses needed for the regulation of cellular homeostasis. However, regulations that specifically activate mTORC1 versus mTORC2 exist, i.e., energy depletion that regulates the AMP-activated protein kinases (AMP) upregulates mTORC2 via RICTOR phosphorylation (77). RICTOR (rapamycin insensitive companion of mTOR) selectively binds mTORC2, as is the case of AMPK activation, mTORC2 when bound to phosphorylated RICTOR and AMPK is catalytically active and upregulates AKT (protein kinase B) to block apoptosis under conditions of acute energy depletion (78). RAPTOR (rapamycin sensitive protein of mTOR) selectively binds and activates mTORC1 catalytic activity and directs mTORC1 sub-cellular localization, i.e., to the lysosome. In addition to RICTOR and RAPTOR binding, additional modes of regulation exist and have been comprehensively reviewed (77). The schematic in **Figure 3** is a simplified version of AMPK involvement in mTOR regulation, including AMPK-mediated negative (78) and positive regulation of mTORC complexes (77).

### 4.5 Analyses of autophagy levels and function

Measurement of LC3-II puncta utilizing imaging of fluorescence signal as a result of GFP- or antibody-tagging of LC3-II and comparative immunoblotting assays for both p62 and LC3-II levels often serve as a proxy for the quantification of autophagosome formation and functional autophagic flux (68, 79). The accuracy of such measurements often requires the administration of a lysosomotropic agent, such as chloroquine (CQ), which raises the pH of the autolysosome, impairing its catalytic activity and inhibiting autolysosomal flux. CQ, HCQ, and NH_4_CL cause alkalinization of lysosomes and autolysosomes and impair autophagic flux, while bafilomycin, an ATPase inhibitor, blocks fusion of the autophagosome with the lysosome (80). Each of these small molecules impairs autolysosomal activity and turnover and results in an accumulation of p62 and LC3-II. This approach allows the steady-state levels of these proteins to be determined and compared to their levels in cells in which autolysosomal flux has not been blocked. This comparison identifies changes in autophagosome function (flux) that occur because of growth conditions, antineoplastic treatments, and/or disease processes (68). Alternative lysosomotropic agents have also garnered recent interest. Notably, Lys05 has been described to be a more potent inducer of lysosomal alkalinity, and therefore a more potent disruptor of autolysosome function (81). To date, only CQ and HCQ have been used in clinical trials to determine contributions of autophagy to disease processes, including cancer.

Besides the use of lysosomotropic agents, various autophagy-targeting molecules have been used such as ULK inhibitors, PI3K inhibitors, VPS34 inhibitors, ATG inhibitors, and lysosome inhibitors (80). Instead of leveraging the pH specificity required for autolysosomal turnover/flux, these molecules instead target various stages of autolysosome development as early as nucleation of the initial phagophore (as seen with ULK and PI3K inhibitors). In addition to chemical targeting, most studies incorporate siRNA or shRNA knockdown of selective ATG genes to inhibit autophagic activity (82). Autophagy targeting in cancer cells is often necessary to determine if autophagy induction by antineoplastic treatments is cytoprotective (promotes cell survival) or cytotoxic (death- inducing), and in some cases, nonprotective. These modes of autophagy have been recently reviewed and are often cell and context dependent (83). In fact, driving autophagy to destroy the cancer cell (referred to as autophagic cell death or ACD) is a potential avenue of targeted therapy, considering that apoptosis is defective in most cancer cells. In a recent review (84) the following criteria were emphasized for a death mechanism to be considered autophagic cell death: 1) autophagic flux is elevated, not inhibited by the therapy; 2) inhibition of autophagy (pharmacological or genetic) blocks treatment-induced cell death; and 3) cell death occurs independent of other types of programmed cell death, i.e. apoptosis. However, there are reports in which autophagy and apoptosis occur in parallel in cancer cells and the inhibition of the autophagic machinery suppresses the intrinsic pathway of apoptosis. An example of this scenario is described in studies showing that induction of autophagy by bromodomain protein (BET) inhibitors is critical for subsequent caspase 3-dependent apoptosis (85). It should be noted that in addition to pro-survival and cytodestructive (cytotoxic) autophagy, two additional functional forms of autophagy have been identified and recently reviewed (86). These include nonprotective autophagy that plays no apparent role in response to antineoplastic agents (i.e., chemotherapy or radiation), and cytostatic autophagy that sustains prolonged growth inhibition, reducing clonogenic survival (loss of reproductive capacity) without induction of apoptotic or necrotic cell death. Whether these forms of autophagy contribute to breast cancer cell dormancy or dissemination (metastasis) in patients has not been determined.

## 5 Autophagy and breast cancer

Importantly, autophagy and/or senescence induction have been identified as a pro-survival response to most antiestrogen treatments and molecular targeting currently being used for the treatment of ER^+^ breast cancer. Although senescence was originally identified as a terminal state of cancer cells, recent studies have highlighted that senescence can be reversible, and thus, is a potential mechanism breast cancer cells can utilize to escape antiestrogen-induced and chemotherapy-induced stress (87–89). Thus, these “survival” response pathways often precede the genetic and epigenetic alterations required to escape cytostasis (likely contributing to tumor dormancy) and activate a functional cell cycle, with subsequent breast cancer cell proliferation. Further, pre-clinical *in vitro* and *in vivo* studies demonstrated that autophagy promotes the survival of dormant breast cancer cells prior to metastatic tumor recurrence (90). Therefore, we will next discuss the evidence that autophagy is pro-survival in antiestrogen-challenged breast cancer cells.

### 5.1 SERM and SERD therapy and pro-survival autophagy

The adaptive pathway of autophagy appears critical for the development of resistance to SERMs (91–93), SERDs (94), and possibly aromatase inhibitors (95). In initial studies, ER^+^ MCF-7 breast cancer cells were exposed to increasing concentrations of 4-hydroxytamoxifen (4-OHT); 4-OHT is an active metabolite of tamoxifen typically used for cell culture studies. MCF-7 cells undergoing 4-OHT selection showed higher levels of autophagy as evidenced by electron microscopy and LC3-II levels determined by Western blotting (92). After several months of selection, the surviving cell population was resistant to tamoxifen and 4-OHT, expressed high levels of autophagosomes, and showed increased apoptosis if autophagy was targeted with CQ, bafilomycin, or siRNA targeting of *Atg6* (Beclin) in the 4-OHT selected breast cancer cells (92). In a parallel study, the Gorski laboratory showed that autophagy inhibition of tamoxifen treated T-47D, MCF-7, HER2/Neu overexpressing, tamoxifen resistant breast cancer cells induced mitochondrial depolarization and apoptosis (96). These initial observations were expanded in subsequent studies conducted *in vitro* (97–101) and *in vivo* (in breast tumor bearing mice) (91) that further identified a cytoprotective role of autophagy in SERM (tamoxifen) and SERD (faslodex) treated breast cancer cells. In some of these later studies, specific mechanisms or molecular regulators of autophagy have been implicated in the pro-survival process such as upregulation of glucose regulated protein 78 (GRP78) (100, 102), induction of lysophagy to eliminate tamoxifen-mediated damaged lysosomes (101), and overexpression of glucose transporter 1 (GLUT1) and LC3B in MCF-7 tamoxifen resistant cells (MCF-7/TAMR-1) and in tamoxifen resistant clinical samples of breast cancer (98). Further, downregulation of GLUT1 sensitized MCF-7/TAMR-1 cells to tamoxifen and decreased autophagic flux. These data link autophagy induction to tamoxifen resistance and identify GLUT1 as a potential molecular target to delay the development of endocrine resistance.

### 5.2 AMPK and antiestrogen-induced autophagy versus cell death

The relationship between tamoxifen-induced autophagy and antiestrogen resistance also involves the upregulation of AMPK. As discussed above (Section 4.4), AMPK is the major sensor of energy in cells. When ATP levels are low, AMP and/or ADP bind to AMPK. This binding results in allosteric modification and AMPK can then be phosphorylated by liver kinase B1 (LKB1) and other kinases (103). Typically, LKB1 and AMPK are considered tumor suppressors; LKB1-mediated AMPK activation induces autophagy that acts in an anti-tumor manner (reduction of tumor growth). However, AMPK also senses ROS and responds by upregulating multiple antioxidant genes, including Catalase, Sod1, Sod2, and Ucp2 (104). In this regard, AMPK activation would protect breast cancer cells from ROS-induced death. This protective action of AMPK, is one example of the double-edged sword described for the roles of autophagy in cancer, in which the pro-survival versus anti-tumor role of autophagy is context and tumor stage dependent (105).

The Clarke laboratory first showed that TSC2/AMPK mediated mTOR inhibition occurred in antiestrogen treated sensitive and resistant breast cancer cells (100). Further clarification of the role of AMPK comes from studies by the Koumenis’ laboratory in which genetic approaches were utilized to selectively downregulate the AMPKα1 and AMPKα2 isoforms (106). Results from this study indicated that AMPKα1 is required for tamoxifen- induced cytotoxicity, while AMPK α2 mediates tamoxifen-induced autophagy. In subsequent clinical studies, downregulation of AMPKα1 was identified in advanced breast cancer and correlated to metastasis and a poor clinical outcome (107). It is interesting to speculate that AMPKα2, which was not downregulated in this cohort of patients, facilitated the aggressive nature of their breast cancers via its role in mediating autophagy. Clearly, these studies underscore the need to analyze the role(s) of the AMPK isoforms in the response of breast cancer cells to all antiestrogen treatments, and to determine if selective blockade of AMPKα2 is a viable molecular approach to combat antiestrogen resistance and delay relapse.

### 5.3 Aromatase inhibitor therapy and pro-survival autophagy

Exemestane, a steroidal AI, also has been shown to induce autophagy in aromatase expressing cells MCF-7 cells (designated MCF-7aro) (108). Aromatase inhibition blocked the activity of the mTOR, a major negative regulator of the autophagy pathway in normal and cancer cells (**Figure 2**), resulting in the activation of AMPK. AMPK is the key energy sensor in mammalian cells and is activated when energy (ATP levels) decreases and AMP levels increase (109). AMPK, once activated, typically inhibits the mTOR complex which is active under nutrient-rich conditions and promotes cell growth (78). Notably, however, nonsteroidal AIs (Letrozole and Anastrozole) did not increase LC3-I/II or suppress SQSTM1/p62 levels in cells, indicating that these treatments may not induce autophagy in breast cancer cells (73). In contrast, Letrozole and Anastrozole increased senescence in the MCF-7aro cells, with measurable upregulation of Yippee-like 3 (*YPEL3)*, a gene associated with tamoxifen induced senescence (110). In addition, in a hormone rich environment, the non-steroidal AIs up-regulated ERα while blocking estrogen signaling; whereas Exemestane downregulated ERα. Of potential clinical relevance, in hormone-depleted conditions, a crosstalk between AR and ERα was identified that enhanced the estrogenic effects of Exemestane, suggesting caution when utilizing Exemestane as a 2^nd^ line endocrine treatment (110). These differential outcomes mediated by the AIs are consistent with distinct facets to their mechanisms of action in breast cancer cells and highlight the need for more detailed studies.

### 5.4 Breast cancer clinical trials and autophagy inhibition

Efforts to target autophagy to increase the efficacy of antineoplastic drugs and reduce cancer cell viability have recently been reviewed (111). In general, these trials utilize the lysomotropic agents hydroxychloroquine (HCQ) and CQ, the only two FDA-approved drugs for autophagy inhibition, and can be accessed at clinicaltrials.gov. Ongoing clinical trials with ER^+^ breast cancer in which HCQ and CQ are being combined with standard of care endocrine therapy, an antiestrogen + CDK4/6i, or chemotherapy are listed in **Table 2**. Published results from a randomized, double-blind clinical trial (NCT02333890) showed no measurable antitumor effects of CQ (500 mg daily) delivered as a single agent for up to 6 weeks prior to surgery, while toxicity was observed in this pre-operative setting (112). These results, although disappointing, do not necessarily predict outcome when HCQ and CQ are combined with other treatment modalities that induce pro-survival autophagy in ER^+^ breast cancer cells. In other cancers, HCQ and CQ combined with chemotherapy or radiation has shown measurable clinical benefit (113). Also, a recently completed trial NCT01446016 in which taxane and taxane like chemotherapeutics (Paclitaxel, docetaxel, abraxane, & ixabepilone) were combined with HCQ showed efficacy for advanced and metastatic breast cancer with prior anthracycline-based chemotherapy (114). The ongoing clinical trials in which CQ or HCQ are combined with antiestrogens and CDK4/6i’s (**Table 1**) are critical and should better define a role for autophagy in breast cancer survival. One caveat that must be considered is the sequencing of CQ/HCQ relative to the other drugs being delivered. It is possible that CQ and HCQ are more effective following induction of autophagy by the standard-of-care therapy. Also, if autophagy contributes to the anti-tumor aspects of antiestrogens and CDKi’s, the timing of targeting may be critical to elicit an effective response. The complexity (yin and yang) of autophagy in cancer cell biology has recently been reviewed (115).

**Table 2:** List of active clinical trials involving hydroxychloroquine or chloroquine treatment of breast cancer in the US.

NCT (Study Name)	Intervention	Phase	Primary Endpoint
NCT03774472: Hydroxychloroquine, Palbociclib, and Letrozole Before Surgery in Treating Patients with Estrogen Receptor Positive, HER2 Negative Breast Cancer	HCQ+ Pal+ LET	II	Evaluate the efficacy and safety of HCQ combined with PAL+LET.
NCT04523857: Abemaciclib or Abemaciclib and Hydroxychloroquine to Target Minimal Residual Disease in Breast Cancer (ABBY)	Abema + HCQ	II	Evaluate use of HCQ +Abema to target disseminated bone tumor cells.
NCT04841148: Avelumab or Hydroxychloroquine With or Without Palbociclib to Eliminate Dormant Breast Cancer (PALAVY)	HCQ Avelumab Pal+Avelumab Pal+HCQ	II	Determine efficacy of HCQ or Avelumab in eliminating bone marrow disseminated tumor cells.
NCT03032406: CLEVER Pilot Trial: A Phase II Pilot Trial of Hydroxychloroquine, EVErolimus or the Combination for Prevention of Recurrent Breast Cancer	HCQ Eve	II	Evaluate the feasibility of administering HCQ, EVE or the combination in patients who have completed primary therapy for breast cancer and harbor bone marrow disseminated tumor cells.
NCT05953350: A Phase Ib/II Study Confirmed Inhibition of Autophagy Synergizes Anti-tumor Effect of High Dose CDK4/​6i	Pal+ HCQ	II	Explore the safety and antitumor efficacy of different doses of Palbociclib in combination with HCQ

NCT, ClinicalTrials.gov identifier; HCQ, Hydroxychloroquine; Eve, Everolimus; Pal, palbociclib; Let, letrozole; Ful, fulvestrant; Abema, abemaciclib.

## 6 Senescence and breast cancer cell survival

Senescence induction is a mode of breast cancer survival that is garnering increased attention due to studies suggesting that reversible senescence may function as a mechanism of tumor dormancy (88, 116–119). Although the relationship between senescence and antiestrogen resistance is not adequately defined, senescence is known to support dormant tumor cell survival (120), contribute to apoptosis resistance (121), cellular stemness, tumor aggressiveness (122), and the metastatic niche (123). Thus, survival by senescence potentially provides breast cancer cells an opportunity to acquire genetic and epigenetic alterations that allow re-entry into the cell cycle, proliferation, and ultimately increase dissemination (metastatic spread). It has been documented that breast cancer cells enter senescence in response to chemotherapy (124) and radiation therapy (125). In this section, we will describe the senescent phenotype and review data available supporting the role for senescence in breast cancer survival in response to antiestrogen + CDKi combination therapy.

### 6.1 The therapy induced senescence phenotype

Senescence has long been typically regarded as an irreversible exit from the cell cycle; this view is based on initial studies utilizing non-transformed fibroblasts (126, 127). The senescent state is identified as stable and durable, with senescent cells remaining viable and metabolically active, but unresponsive to mitogenic drivers. There are three general forms of senescence: replicative senescence, oncogene-induced senescence, and,our focus in this chapter, therapy-induced or accelerated senescence. Accelerated senescence is induced by antineoplastic agents such as chemotherapy, radiation, and endocrine therapy (117, 128). Recent findings, including from our laboratory (129–132), have identified a reversible type of therapy-induced senescence, termed reversible senescence, during which senescent cells escape growth arrest, re-enter the cell cycle, and proliferate (133).

During senescence-mediated growth arrest, tumor cells can secrete a wide range of cytokines, chemokines, growth factors, and matrix remodeling factors, known collectively as senescence-associated secretory phenotype or SASP factors (134). SASP factors alter the local tissue environment (135, 136) and impair the innate as well as the adaptive immune response (135, 137). These actions prevent the elimination of senescent cells, resulting in their accumulation in tissues, which ultimately contributes to chronic inflammation, cancer progression and metastasis (138, 139). However, recent studies by the Lowe laboratory provide provocative evidence that senescence in advanced cancers may also render tumor cells more visible to the adaptive immune system (137). This antitumor immunity mediated by senescent cells involves heterotypic signaling interactions and is dependent on the upregulation of IFNγ receptor and can be inhibited without effect on the senescence phenotype, including the SASP. Whether this role of senescent cells can be exploited to effectively eradicate non-senescent tumor cells remains to be determined.

The most common SASP factors analyzed to identify the senescence phenotype are IL-1α, IL-1β, IL-6, IL-8, as well as MMP. In addition to the SASP and the durable growth arrest, the senescent phenotype consistently shows increased β-galactosidase (SA-β-Gal) (140); SA-β-Gal expression, typically detected by a histochemical assay, is considered the gold standard for senescence detection (141). Additional approaches to detect SA-β-Gal expression include the flow cytometric quantification of C12FDG (5-dodecanoylaminofluorescein di-β-D-Galactopyranoside) fluorescence, a metabolite for SA-ß-Gal (142). Senescent cells are further characterized by the accumulation of reactive oxygen species (ROS) (143), an enlarged and flattened morphology, chromatin rearrangement known as senescence-associated heterochromatic foci (SAHFs), and nuclear foci termed DNA segments with chromatin alterations reinforcing senescence (DNA-SCARS) (144). Additional markers of senescence can include Yippee-like-3 (YPEL3) expression, a tumor suppressor that is induced by DNA damage (145, 146) and downregulation of Lamin B1 expression (147). It is important to note that an accurate detection of senescence induction is dependent on a combination of these markers.

### 6.2 Senescence targeting: senostatics, senomorphics, and senolytics

An overarching goal in cancer treatment is the promotion of apoptosis or other forms of cell death in the tumor cells. In particular, most chemotherapy drugs rely on the induction of the intrinsic pathway of apoptosis to kill cancer cells (148). However, a major characteristic of senescent cells is resistance to chemotherapy induced apoptosis, which is usually attributed to the upregulation of the anti-apoptotic proteins BcL_xl_, BcL_W_ and BcL_2_ (121, 149, 150), which may ultimately contribute to tumor recovery (escape from dormancy). Therefore, targeted therapies are being studied that can eradicate senescent cells or suppress their recovery. Three major types of senescence-targeted therapies are utilized in pre-clinical studies; senostatics, senomorphics, and senolytics (151–157).

Senostatics do not kill senescent cells but extend the growth arrest state, inhibit paracrine signaling, and thus block the recovery of the senescent population. Antioxidants or NF-κB inhibitors have been investigated as senostatics (143, 158), in addition flavonoids, polyphenols and other phytochemical molecules may have potential senostatic activity (159). Senomorphics block senescence-mediated signaling via suppression of SASP expression by a plethora of molecular pathways including NF-κB, mTOR, IL-1α, and p38 MAPK (159). In contrast, senolytics eliminate senescent cells via targeting critical proteins involved in pro-survival and anti-apoptotic mechanisms, most prominently the Bcl-2 family of proteins (159). Various senolytics, including ABT-263, interfere with the antiapoptotic proteins Bcl-2, Bcl-w, and Bcl-X_L_ (160) and have been investigated for their ability to eliminate senescent tumor cells that are induced by etoposide, doxorubicin, cisplatin, and radiation. These senolytics interfere with the interaction between BCL_XL_ and BAX (and potentially other pro-apoptotic BH3 members, i.e., Bid, Bad, and Bim) in cancer cells (154, 161, 162). Overall, studies aimed at eradicating senescent cells and/or their recovery are highly relevant to improving therapeutic approaches aimed at eliminating cancer cells, particularly considering the multiple tumor- promoting roles ascribed to senescence (escape from dormancy, SASP, apoptosis resistance, and increased stemness).

### 6.3 Senescence and anti-estrogen therapies

Several studies have investigated senescence and its relationship with tamoxifen, the most commonly used SERM for the treatment of ER^+^ breast cancer. Lee et al. showed that tamoxifen treatment at 10μM (concentration that exceeds the clinically achieved levels) for 4 days induced senescence in MCF-7, and T-47D cell populations (163). The induction of senescence was identified by SA-β-Gal staining (25% higher than vehicle-treated MCF-7 cells, and 20% higher than vehicle treated T-47D cells), and the upregulation of p53 and p21. In addition, the level of reactive ROS was elevated in tamoxifen-treated MCF-7 and T-47D cells. Treatment with either NAC, a commonly used ROS scavenger, or apocynin, an inhibitor of NADPH oxidase involved in superoxide anion production (164), suppressed SA-β-Gal activity and p53 protein expression in MCF-7 cells. NAC treatment also suppressed ROS production in T-47D cells known to express mutant p53. Mechanistically, this study showed that tamoxifen inhibited protein kinase CK2 catalytic activity, a Ser/Thr kinase known to catalyze the phosphorylation of large numbers of both cytoplasmic and nuclear proteins that are overexpressed in breast cancer cells (165, 166) (165). CK2α (CK2 catalytic subunit) overexpression suppressed tamoxifen-induced senescence in MCF-7 cells, reduced p53 and p21 levels, as well as ROS production. Thus, Lee et al. proposed that tamoxifen induced senescence is CK2 dependent and mediated via an ROS–p53–p21-dependent pathway in breast cancer cells. Whether senescence induction in these breast cancer models involved mTORC1 or mTORC2 action was not investigated. However, Jung et al. (167) have identified each mTOR kinase as being able to bind to p53 and induce a senescence phenotype, albeit the studies were performed in a PTEN-deficient MCF-7 cells and mouse embryo fibroblasts with dysregulated PI3K/AKT signaling.

Increased expression of YPEL3, a p53-regulated protein known to induce senescence (145) also has been proposed to induce senescence in antiestrogen treated MCF-7 cells (110). Tamoxifen treatment, at a concentration of 0.5 μM for six days, resulted in a 2.3-fold induction of YPEL3 mRNA expression compared to its levels in control cells; this was also associated with a 35% increase in SA-β-Gal positive cells above basal levels in control cells, indicative of senescence induction. These results were not observed when YPEL3 expression was inhibited with shRNA targeting, confirming the role of YPEL3 in tamoxifen induced senescence. These *in vitro* results indicate an association between tamoxifen treatment and senescence induction, but need to be substantiated in breast tumor bearing animal models in which clinically relevant concentrations of tamoxifen are achieved, i.e. as high as 1.0 µM in patient tissues and 0.3 µm in serum (168).

### 6.4 CDK4/6i’s, breast cancer senescence, and mTORC1

The most frequently utilized CDKi, usually in combination with either fulvestrant or letrazole, is palbociclib (130, 169). Palbociclib is a potent inducer of the senescence phenotype; for example, Jost et al. showed that palbociclib treatment (2uM for 10 days) increased the percentage of C12FDG, an established marker of senescence, by 20-30%) in MCF-7 cells (170). Maskey et al. also investigated the relationship between senescence and palbociclib in ER^+^ breast cancer using MCF-7, T- 47D and the ER^+^ breast cancer cell line CAMA-1 (171). These studies indicated that palbociclib arrested the ER^+^ cell populations in G1 phase, increased cell size, and induced β-galactosidase expression (increased SA-β-Gal staining). These changes occurred in a retinoblastoma (Rb) dependent manner; Rb knockdown via shRNA targeting in CAMA-1 cells prevented palbociclib-induced growth suppressive effects. Importantly, the palbociclib-induced senescence in MCF-7 and T-47D cells was reversible. Removal of palbociclib after 6 and 14 days for both cell lines resulted in a breast cancer cell population with normal morphology, measurable proliferation, and reduced SA-β-Gal activity. CAMA-1 cells also recovered their proliferative capacity after 6 days of treatment. However, after 14 days of treatment the CAMA-1 cells remained enlarged, growth arrested, SA-β-Gal positive, and ultimately transitioned to cell death. Palbociclib upregulated expression of SASP genes to a higher level in CAMA-1 cells, after 14 days of treatment, than detected in palbociclib-treated T-47D and MCF-7 cell populations. Based on these results the authors proposed that CAMA-1 cells entered a complete/irreversible state of senescence after 14 days of palbociclib treatment, while T-47D and MCF-7 entered an incomplete (reversible) senescence.

In the study conducted by Maskey et al. the underlying mechanism(s) of palbociclib-induced senescence were also addressed (171). Treatment for either 6, 10 or 14 days was shown to decrease phosphorylation of downstream components of the mTORC1 pathway (4EBP1, S6K1 and S6RP), suggesting that mTORC1 activity was downregulated in MCF-7 and T-47D cells. In contrast, palbociclib treatment for 10 or 14 days of CAMA-1 cells did not affect the levels of the mTORC1 substrates, suggesting a relationship between mTORC1 and irreversible senescence in response to the long-term palbociclib treatment. Importantly, blocking mTORC1 action with rapamycin, a small molecule inhibitor of mTORC1 (172), or by shRNA targeting of Raptor, a positive modulator of mTORC1 complex activity (173), prevented palbociclib-induced irreversible senescence in CAMA-1 cells. In addition, in MCF-7 cells, CRISPR-mediated genetic depletion of tuberous sclerosis complex 2 (TSC2), a negative regulator of mTORC1 (174), in combination with palbociclib for 14 or 21 days induced sustained growth arrest, flattened morphology, and intense SA-β-Gal staining as compared to the control cell population. The results from these pharmacologic and genetic approaches provide strong evidence that mTORC1 is involved in palbociclib-induced cellular senescence (171). Of note, autophagy relative to senescence induction was not evaluated in these studies, nor was a potential role of mTORC2 addressed. However, Bernard et al. (175) have determined that prolonged autophagy can drive senescence through ROS-mediated mTORC2 activation in fibroblasts. This important link between autophagy and senescence was established, in part, by siRNA targeting of Rictor, an essential component of the mTORC2 complex. Suppression of mTORC2 activity decreased the expression of the cyclin dependent kinases inhibitors CDKN1A and CDKN2A, identified mediators of senescence. Whether mTORC2 activity plays a role in CDKi- and/or antiestrogen-induced senescence should be considered.

Analysis of palbociclib resistant MCF-7 and T-47D cell lines also has provided mechanistic information about senescence. The palbociclib resistant (ER+) breast cancer cells overexpressed cyclin E and c-Myc, with loss of RB function as compared to their parental counterparts (176). In this study, the relationship between palbociclib sensitivity and cell cycle-related gene expression was further analyzed in 38 breast cancer cell lines from the Cancer Cell Line Encyclopedia (CCLE) data base (177). A strong correlation was identified between low palbociclib sensitivity and high expression of the cell proliferation promoting moiety, cyclin E. The analysis of 11 pleural effusion samples from hormone-positive breast cancer patients showed a similar correlation, specifically cyclin E overexpression, in addition to low RB status correlated with palbociclib resistance. These studies therefore identified the cyclin E-CDK2 signaling node as a molecular target to potentially increase the sensitivity of the resistant cells to palbociclib. CDK2 knockdown, via siRNA targeting, in combination with palbociclib synergistically reduced the cell proliferation and viability of both MCF-7 wild type cells and the resistant cell line as compared to each treatment alone. The combined treatment suppressed c-Myc (and phosphorylated c-Myc) and hTERT, a downstream target of c-Myc (178) and a key regulator of senescence (179), which were upregulated in the palbociclib resistant cells, more effectively than CDK2 inhibition alone. Importantly the combination therapy compared to single agent therapy induced higher SA-β-Gal activity (90-100% for the combination, CDK2 inhibition alone ~50%, and palbociclib alone ~25%) in palbociclib resistant MCF-7 cells. These results were validated *in vivo* using MCF-7 Palboiclib-resistant xenograft mouse models; here the combination of CDK2 inhibition, via siRNA targeting, and palbociclib resulted in a more significant tumor regression as compared to each drug alone. Furthermore, western blot analysis showed inhibition of the phosphorylated form of c-Myc, a reduction in hTERT, and increased levels of cleaved caspase-3 (marker of apoptosis). Similar results were generated by Pogacar et al. (180), using the CDK2 inhibitor, indisulam, in triple-negative breast cancer, where the combination of palbociclib and indisulam resulted in sustained senescence induction.

### 6.5 CDKi’s: cross talk between senescence and autophagy

An important unanswered question is the relationship between autophagy and senescence in breast cancer treated with antiestrogens and/or CDK inhibitors. A recent study by Vijayaraghavan et al. addressed the relationship between autophagy and senescence in ER^+^ breast cancer cells exposed to palbociclib (52). Palbociclib treatment for 6 days significantly inhibited the growth of the ER^+^ MCF7, T-47D, and ZR75-1 cell lines, in a time- and dose-dependent manner. Palbociclib treatment at doses of 1 μM or less caused reversible G1 arrest and growth inhibition in MCF-7 and T-47D cells, together with increased SA-ß-Gal activity. Treatment with higher doses of palbociclib (>2.5 μM) resulted in irreversible inhibition of growth, without apoptosis induction. Importantly, the induction of senescence in response to palbociclib (1 μM for 6 days) was coupled with autophagy induction as shown by increased monodansylcadavarine (MDC) staining, upregulation of LC3B-II and other key autophagy related proteins (Atg-7, Beclin-1, BNIP3), a reduction in BCL_2_ and p62, and accumulation of double-membrane electron-dense autophagosomes and GFP-LC3 puncta. Autophagy inhibition, genetically via Beclin1-targeted shRNA or ATG5-targted shRNA, sensitized MCF-7 and T-47D to palbociclib, with induction of irreversible G1 growth arrest and elevated senescence. The combination of HCQ and palbociclib also resulted in sustained cellular growth inhibition, irreversible G1 arrest, a significant increase in ROS levels and cellular senescence, without inducing apoptosis. Similar results were obtained with other pharmacological autophagy inhibitors, including Lys05, CQ, and bafilomycin A1, when combined with palbociclib. These results were recapitulated *in vivo* using orthotopic xenografts treated with increasing concentrations (25–150 mg kg−1 per day) of palbociclib for 7 days. Palbociclib treatment reduced the tumor volume, increased ROS production, increased SA-ß- Gal activity, and upregulated senescence proteins in a dose dependent manner as showed by reverse-phase protein array (RPPA). Western blot, RPPA and TEM analyses of the tumors treated with palbociclib 25 mg kg−1 per day, showed higher levels of LC3B-II and Atg-7, a reduction of p62, and the presence of double-membrane electron-dense autophagosomes, suggesting autophagy induction. Importantly, treatment with a combination of HCQ and palbociclib showed a significant reduction in tumors volume as well as increasing ROS production together with increased SA-ß-Gal activity. Similar results were obtained with abemaciclib and ribociclib in that HCQ enhanced the anti-proliferative response of these CDK inhibitors with apoptosis induction in MCF-7 and T-47D cells. These data show that autophagy and senescence may occur in parallel and provide strong evidence that autophagy inhibition can serve as a senostatic agent/approach.

### 6.6 Senescence and epigenetic dysregulation

The dysregulated transcription machinery in cancer cells is gaining traction as a potential targets in cancer biology, specifically the potential incorporation of epigenetic regulators into therapy (181). The epigenetic machinery contributes to gene regulation, consisting of more than 600 epigenetic regulators (genes), controlling reading, and erasing histone and DNA modifications (182). However, dysregulated transcription machinery contributes to overexpression of many genes, including oncogenes, which contribute to tumorigenesis. Furthermore, epigenetic machinery is associated with the senescence phenotype. Senescent cells express many features under epigenetic regulation including senescence-associated heterochromatin foci (SAHF) (183). SAHF consist of a condensed chromosome enriched with histone modifications, including H3K9me3 as well as H2AX phosphorylation (184), and proteins associated with epigenetically silenced genes such as members of the E2F family (185). Further, SAHF suppress the expression of proliferative genes, including cyclin A, mediating cellular growth arrest, the common feature of senescence (186). The SASP is also regulated via various epigenetic regulators including high-mobility-group protein B2 (HMGB2) (187, 188), mixed-lineage leukemia 1 (MLL1) (189), disruptor of telomeric silencing 1-like (DOT1L) (190), NAD-dependent deacetylase sirtuin-1 (SIRT-1) (191) and BRD4 (192).

Super-enhancers are one of the dysregulated epigenetic elements in cancer cells that are identified as potential molecular targets. Super-enhancers are a cluster of regulatory regions, specifically enhancers, with unusually strong enrichment for the binding of transcriptional coactivators, readers, and transcription factors (193). Super-enhancers can be essential oncogenic drivers, leading to high expression of many oncogenes (194). Different families of transcription factors occupy super-enhancer sites; prominently among which is the bromodomain and extraterminal domain (BET) family. The BET family consists of four conserved mammalian members (BRD2, BRD3, BRD4, and BRDT) that regulate the expression of many genes and signaling pathways and BET family members are commonly dysregulated in cancer cells (195). The BET family members identify and bind to acetylated histones, acting as epigenetic readers for histone acetylation, and facilitate the recruitment of transcriptional regulatory complexes to chromatin (196). Further, chromatin remodeling during senescence results in super-enhancer development at SASP genes, increasing SASP expression. H3K27 acetylation as well as BRD4 recruitment are crucial events for SASP expression (192). Therefore, BET inhibitors have emerged as a promising class of anticancer drugs with many agents developed to be utilized in combination with various chemotherapeutic modalities (197). The efficacy of BET inhibitors as an approach to target senescence has been investigated in a number of studies, including those from our laboratories with senolytic as well as senomorphic activity being demonstrated (85, 130, 192). The relationship between the BET family and autophagy has recently has been reviewed by our laboratory (198).

### 6.7 BET inhibitors and targeting senescence in ER (+) breast cancer

Recently, our research group (130) investigated the relationship between senescence, BET inhibitors and fulvestrant plus palbociclib in the ER+ MCF-7 and T-47D breast cancer cell lines. This study showed that palbociclib treatment, at the concentration of 1µM for 6 days, induced cellular senescence. Growth arrest, increased SA-ß gal staining, and quantification of SA-ß-Gal by assaying for C12FDG with flow cytometry were demonstrated in the palbociclib-treated cell populations. The induction of senescence was further confirmed by assessing SASP factors, IL6, IL8 as well as MMP3, using Q-PCR. Similar results were obtained using the combination of palbociclib with fulvestrant, which would be the most likely breast cancer combination treatment in the clinic. We identified escape from the growth suppressive effect of the combined treatment (fulvestrant plus palbociclib) between days 8 to 12 days of treatment. In contrast, in Rb-depleted MCF-7 and T-47D cells, the combination treatment for 6 days induced growth arrest, but failed to induce senescence, suggesting that senescence induction is mainly driven by Rb. These results are consistent with the low Rb status in palbociclib resistant MCF-7 and T-47D cells (176, 199). Overall, in ER^+^ breast cancer cells expressing functional Rb, standard-of-care treatment for breast cancer (fulvestrant + palbociclib) induced senescence that was reversible and, therefore, could mediate tumor relapse.

Having established that palbociclib + fulvestrant treatment induced reversible senescence, we next evaluated how the BET inhibitor/degrader ARV-825 affected the senescent state. ARV-825 (50nM for 4 days) suppressed the recovery of the senescent population. Importantly, ARV-825 showed senolytic activity; approximately 50% cell death was identified in the senescent cell population (induced by fulvestrant + palbociclib) as determined by annexin V/PI staining. ARV-825 also suppressed recovery from growth arrest and induced apoptosis in the Rb-depleted cell populations treated with fulvestrant plus palbociclib. Mechanistically, ARV-825 suppressed BRD4 as well as its downstream effector, c-Myc.

Further support for the potential of ARV-825 as a molecular targeting strategy for senescent cancer cells is based on two studies (78): Mo et al. provided evidence that S6K, a downstream target of c-Myc, is involved in clinical expression of CDK4/6 resistance (200); and (76) Wakita et al. determined that ARV-825 eliminates the senescence population in different cancer models, in part, through autophagy induction. In this latter study, ARV-825-induced autophagy was required for the senolytic activity, along with an exacerbation in the number of double strand DNA breaks due to attenuation of nonhomologous end joining (NHEJ) also induced by ARV-825 treatment (85). Collectively, these studies show that CDKi’s induce senescence in breast cancer cells and suggest that BET inhibitors have excellent potential to be used as senostatics or senolytics to delay relapse and/or overcome palbociclib resistance.

## 7 Established mechanisms of antiestrogen resistance

Survival mechanisms, such as autophagy and “reversible” senescence provide a finite amount of time for breast cancer cells to adapt/survive antiestrogen (and CKD inhibitor therapy), during which breast cancer cells are typically growth arrested. During what may be described as a period of dormancy, breast cancer cells potentially undergo genetic and/or epigenetic changes that facilitate escape from growth arrest and expression of estrogen independence and/or antiestrogen resistance. However, studies have not yet identified the biochemical and molecular foundation(s) of this proposed temporal progression to antiestrogen resistance. On the other hand, multiple mechanisms of acquired antiestrogen resistance that contribute to a poor clinical response have been established and involve mutations and/or upregulation of growth factor signaling pathways, cell cycle regulatory proteins, and anti-apoptotic genes that could over-ride dormancy (the autophagic or senescent state). These mechanisms include overexpression of cyclin D (upregulating the cyclin D/CDK4/6 Rb axis) that can now be targeted with CDK4/6 inhibitors as discussed above, constitutive activation and/or gene amplification of receptor tyrosine kinases including members of the epidermal growth factor family (EGFR & HER2/Neu), insulin like growth factor receptor (IGF-1), and fibroblast growth factor receptor (FGFR) and common downstream targets of these receptors (PI3K and AKT), overexpression of the anti-apoptotic protein Bcl2, and upregulation of heat shock factor 1 (HSF1). In addition, antiestrogen resistance can result from mutations, altered expression, and/or amplification of the *ESR1* and *CYP19* genes that encode ERα and aromatase, respectively. Many of these mechanisms have been recently reviewed (201–203); therefore, we will present only a brief synopsis of the available information.

### 7.1 *ESR1* and *CYP19A1* mutations and gene amplification

Typically, ERα is activated by estrogen, facilitating the transcription of genes involved in cell proliferation (204, 205). However, mutations in ESR1 can cause constitutively active ERα which can drive cell proliferation in an estrogen independent manner (204, 205). Mutations in ESR1 are associated with resistance to SERMs (i.e. tamoxifen) and AI therapy (204). For example, expression of ERα with the K303R mutation (a lysine to arginine transition at amino acid residue 303) results in anastrozole resistance (206). This mutant ER is hypersensitive to E2 such that low concentrations of estrogen activate ER signaling, which in turn leads to cell proliferation and circumvents the effect of AIs, ultimately leading to resistance. This mutant ER results from a frequent somatic mutation at nucleotide 908 of *ESR1* (A908G) that is detected in premalignant breast lesions and invasive breast cancers (207, 208). As a consequence of the mutation, increased binding of the mutant receptor with the p85 subunit of phosphatidylinositol-3-OH kinase (PI3K) and constitutive AKT activation occur. Thus, inhibition of the phosphatidylinositol-3-OH kinase (PI3K)/Akt pathway was proposed as a promising therapeutic strategy for hormone-resistant cancers that harbor the K303R mutant ER (206). A recent review highlights the identification of ligand-independent mutations that occur during aromatase inhibitor therapy in metastatic ER^+^breast cancer (209). Sensitive detection methods utilizing patient liquid biopsies have been used to track *ESR1* somatic mutations during tumor progression and this approach (mutation tracking) has the potential to be used to guide sequential treatment options in patients, particularly if it can be applied to circulating tumor DNA (209). With the improvement of sequencing methods, (i.e., deep sequencing and droplet digital PCR) a hotspot for *ESR1* mutations (210, 211) within the ligand binding domain of ER has been identified. Current data identifies mutations in the ER of primary breast tumors, but also indicates that the acquisition of *ESR1* mutations (212) can occur in metastatic breast cancer cells, independent of their presence in the primary tumor. Post-therapy, 20% of patients relapse within 10 years of AI therapy (204, 213, 214). This highlights the importance of understanding and addressing the molecular mechanisms of AI resistance to improve patient outcomes.

The *CYP19A1* gene, encoding for the aromatase enzyme, can also be mutated in breast cancer cells (213). In addition, *CYP19A1* gene amplification has been reported *in vitro* and in patients, attributing to the relapse of approximately 21.5% of patients treated with the reversible AIs letrozole or anastrozole (213). Amplification of *CYP19A1* results in upregulation of aromatase with increased levels of estrogens, which in turn upregulate ligand independent ERα activity and confer resistance to AIs (37, 213). Furthermore, *ESR1* and CYP19A1 appear to co-amplify cooperatively in AI-treated patients as it was found that 32.5% of AI-treated patients had *CYP19A1* amplification, 21.5% presented with *ESR1* amplification, and approximately 66% of breast cancers with *CYP19A1* amplification showed *ESR1* co-amplification (213). These copy number variations are not found in naïve breast cancer cells and appear to be specific to treatment with the reversible AIs (213)(letrozole and anastrozole) in contrast to exemestane treatment.

### 7.2 Aromatase and ERα crosstalk with growth signaling pathways

Beyond genetic aberrations, ER can be activated by upregulated plasma membrane crosstalk with growth factor pathways such as EGFR, epidermal growth factor receptor, epidermal growth factor receptor 2 (HER2), insulin-like growth factor 1 (IGF-1), and fibroblast growth factor receptors (FGFR) which undergo phosphorylation and dimerization and trigger p85, an intracellular regulatory subunit of PI3K/Akt (204). Constitutive activation of the PI3K/Akt/mTOR pathway has been found to not only increase breast cell proliferation and survival, but also induce resistance to AI *in vitro*, particularly to anastrozole and letrozole (204–206). Thus, inhibitors have been developed and are available to target various components of this signaling pathway with the goal of restoring antiestrogen sensitivity in aromatase inhibitor resistant breast cancer cells (1, 204–206). For example, everolimus, an inhibitor of mTOR, in combination with exemestane and ribociclib has shown efficacy for the treatment of advanced ER^+^ HER2 negative breast cancer after progression on treatment with CDK4/6i (215, 216)

HER2, a transmembrane receptor, when activated, initiates a cascade of mitogenic intracellular signaling pathways, including the Raf/MEK/MAPK pathway (217). Activation of Raf/MEK/MAPK1/2 leads to the phosphorylation of ERα (218). This phosphorylation increases the number of available binding sites for estrogen ligands, enhancing ER signal transduction ability (218). Since the ER signaling pathway is paramount to cell proliferation in ER^+^ breast cancers, the overactivation of this pathway contributes to AI resistance. This mode of AI resistance and the various cell culture models utilized to study AI resistance have been reviewed (205). In early studies, MEK1/MAPK1/2 mediated signaling in breast cancer and cross-talk with ERα was also associated with anti-estrogen resistance and a poor prognosis for breast cancer patients (217). Unfortunately, targeting of MEK1/MAPK1/2 signaling in clinical trials (219, 220) has not recapitulated the promising pre-clinical *in vitro* and *in vivo* studies that identified MEK1/MAPK1/2 as a molecular target to block breast cancer cell survival and progression (217, 221–223). Even the combination of a MEK inhibitor with AI did not prove effective as a treatment for advanced-stage breast cancer (224). In a recent study by our laboratory, we identified the upregulation of the EGFR/MEK1/MAPK1/2 signaling axis in an estrogen resistant breast cancer cell model established from MCF-7 cells. In this study, we determined that pro-survival autophagy was attenuating the cytotoxic effects of MEK1 and EGFR inhibitor (selumetinib) treatment (225). Thus, autophagy may be a potential molecular target to improve targeted therapies aimed at the EGFR/MEK/MAPK1/2 signaling node in breast and other cancers.

### 7.3 Heat shock factor 1, ER regulation and antiestrogen resistance

In a recent study (226), HSF1 overexpression was identified in the well-established antiestrogen resistant breast cancer cell models LCC2 and LCC9 that were derived from MCF-7 cells in response to selection with tamoxifen and/or fulvestrant (227, 228). In addition, this study showed that HSF1 regulated ERα expression levels in ER^+^ MCF-7 and T-47D breast cancer cells. HSF1 overexpression induced ERα degradation, decreased ERα-mediated gene regulation, and mediated antiestrogen resistance. Although, overexpression of heat shock factor 1 (HSF1) is associated with treatment resistance and poor prognosis in many cancers (229), HSF1 has not been implicated in antiestrogen resistance prior to this recent study. HSF1 is a molecular chaperone protein and is considered a master regulator of the stress response in mammalian cells and regulates the transcription of genes involved in metabolism, survival, and proliferation (230). Deletion of HSF1 has been reported to suppress the development of breast and other tumors, including pancreatic tumors and tumors of the digestive system (229), and is considered a cancer biomarker and an attractive molecular target (231). Thus, HSF1 inhibitors have been developed (232). Silveira et al. utilized the small molecule inhibitor KRIBB11 to target HSF1. When KRIBB11 was used in combination with tamoxifen or fulvestrant, antiestrogen sensitivity was restored to the antiestrogen resistant LCC9 cells; however, a concomitant restoration in ERα expression was not observed. Thus, in this study HSF1 downregulation resulted in ERα degradation, whereas inhibition of HSF1 transcription activity by KRIBB11 treatment did not reduce ERα expression. Nonetheless, the transcriptional impairment of HSF1 by KRIBB11 was able to sensitize LCC9 cells to antiestrogens. These studies warrant further evaluation of the role of HSF1 in the development of antiestrogen resistance, particularly if one considers the key role that has been reported for HSF1 in coordinating the regulation of components of the heat shock.

## 8 Conclusion

In this chapter we review the concept that endocrine resistance (intrinsic and acquired) as the major obstacle in the clinic to the effective treatment of ER^+^ HER2/neu negative breast cancer and emphasize the need to mechanistically understand how endocrine resistance develops to SERMs, SERDs, and AIs delivered as single agents or in combination with CDKi’s. Toward this goal, we review multiple studies that have identified autophagy, senescence, or both of these adaptive pathways and their roles in supporting breast cancer cell survival in response to antiestrogens and/or CDKi’s. An overview of the saliant features of the central core machinery of the autophagy pathway is provided with a discussion of recent studies identifying AMPK, a major regulator of the mTOR complexes, as a driver of SERM induced autophagy and apoptosis, dependent on the distinct functions of the two AMPK isoforms (AMPKα1 and AMPK α2). Cellular senescence is also reviewed as a response to antiestrogen and CDKi treatment. We posit that targeting these pathways will delay the development of endocrine and CDKi resistance but emphasize that effective targeting may require a sequential treatment approach in which autophagy inhibitors and/or senolytics, senomorphics, or senostatics, are delivered after the antiestrogen plus CDKi treatment has activated/induced these pathways.

In this review, there is minimal focus on the mechanistic relationship between autophagy and senescence in the development and expression of antiestrogen resistance. This is due to the paucity of data in this research area. Thus, detailed mechanistic studies are needed to determine the relationship between autophagy and senescence in the response of breast cancer cells to SERDs, SERMs, AIs and/or CDKi’s. Key questions that need to be answered are: 1) Does autophagy support the metabolism of cells in a state of “reversible” senescence? 2) Can protective autophagy be targeted in a manner that does not interfere with the long-term cytostatic benefits of CDKi’s? 3) How can senescence be most effectively targeted in breast cancer cells undergoing CDKi treatment (in conjunction with the CDKi or after CDKi has induced cellular senescence)? 4) What is the reliability of targeting autophagy and senescence clinically with minimal adverse side effects and, to this end, do we need to develop more selective small molecule inhibitors of autophagy for clinical use? 5) In clinical studies, how can we reliably detect autophagic inhibition in tumor cells? 6) What is a valid marker of the senescence phenoptype that can be readily utilized in the analyses of disseminated tumor cells? Also, if autophagy and senescence serve, in part, as adaptive pathways of survival for breast cancer cells challenged with antiestrogens and/or CDKi’s, then their targeting should be an initial therapeutic approach, not after acquired resistance is expressed. The complexity of the underlying genotypes of acquired antiestrogen resistance include multiple genetic mechanisms and epigenetic changes, reviewed herein, indicating that the breast cancer cells have progressed to a high state of genomic instability following their escape from the cytostatic effects of antiestrogen and/or CDKi therapies. Thus, in breast cancer patients with acquired resistance the most effective approach is personalized medicine/diagnostics which is cost prohibitive for many patients. Evaluation of breast cancer gene signatures is a more generalized approach and cost-effective. Expression profiles predicting intrinsic or acquired resistance to antiestrogens are being utilized; however, profiles are not available to predict intrinsic resistance or the potential to develop resistance to a particular CDKi. Only as we generate accurate and clinically relevant mechanistic information, will signature profiles be developed (for CDKi’s) and improved (for antiestrogens) to more accurately predict appropriate molecular targets and therapies.

## Author contributions

MM: Conceptualization, Writing – original draft. AA: Writing – original draft, Writing – review & editing. LG: Writing – original draft. AE: Writing – review & editing, Writing – original draft. JB: Writing – review & editing. NM: Writing – review & editing. DG: Supervision, Writing – review & editing. PS: Conceptualization, Supervision, Writing – original draft, Writing – review & editing.
